# Supplementary material for: An Integrated Chemical Proteomics Approach for Quantitative Profiling of Intracellular ADP-Ribosylation
Source: Sci Rep. 2019 Apr 30;9:6655. doi: 10.1038/s41598-019-43154-1 (PMC6491589; doi:10.1038/s41598-019-43154-1)
Supplement: Supplementary file 1 — Supplementary Information [file 41598_2019_43154_MOESM1_ESM.pdf]

An Integrated Chemical Proteomics Approach for Quantitative Profiling of Intracellular ADP-Ribosylation

Karunakaran Kalesh[1,2], Saulius Lukauskas[1,3], Aaron J. Borg[4], Ambrosius P. Snijders[4], Vinay Ayyappan[5], Anthony K. L. Leung[5], Dorian O. Haskard[6] and Peter A. DiMaggio[1*]

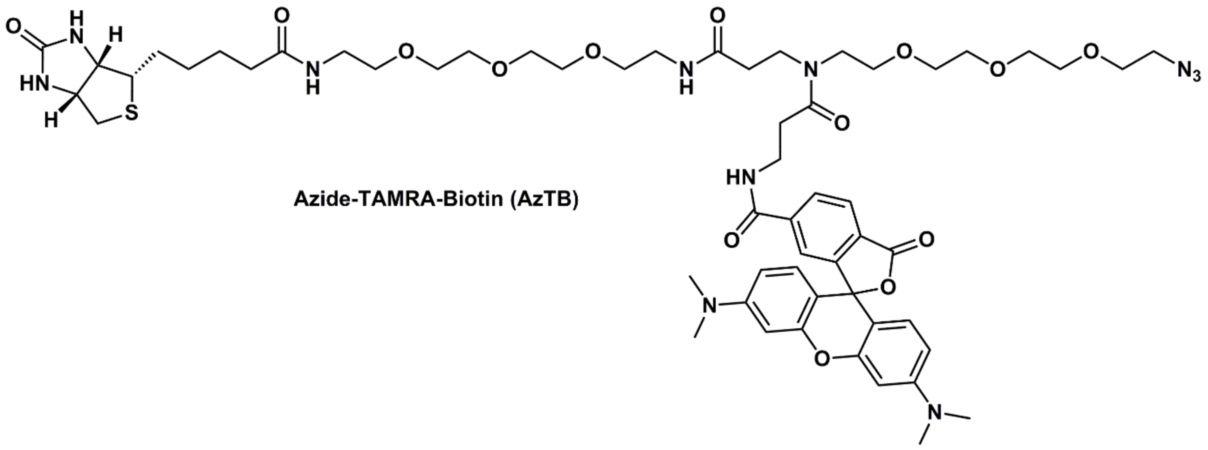

Azide-TAMRA-Biotin (AzTB)

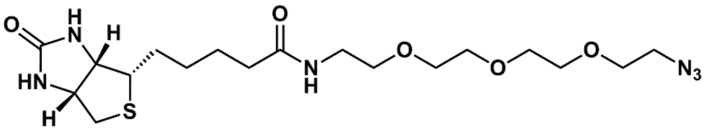

Azide-PEG3-Biotin

Suppl. Fig. 1. Chemical structures of the capture reagents used in this study.

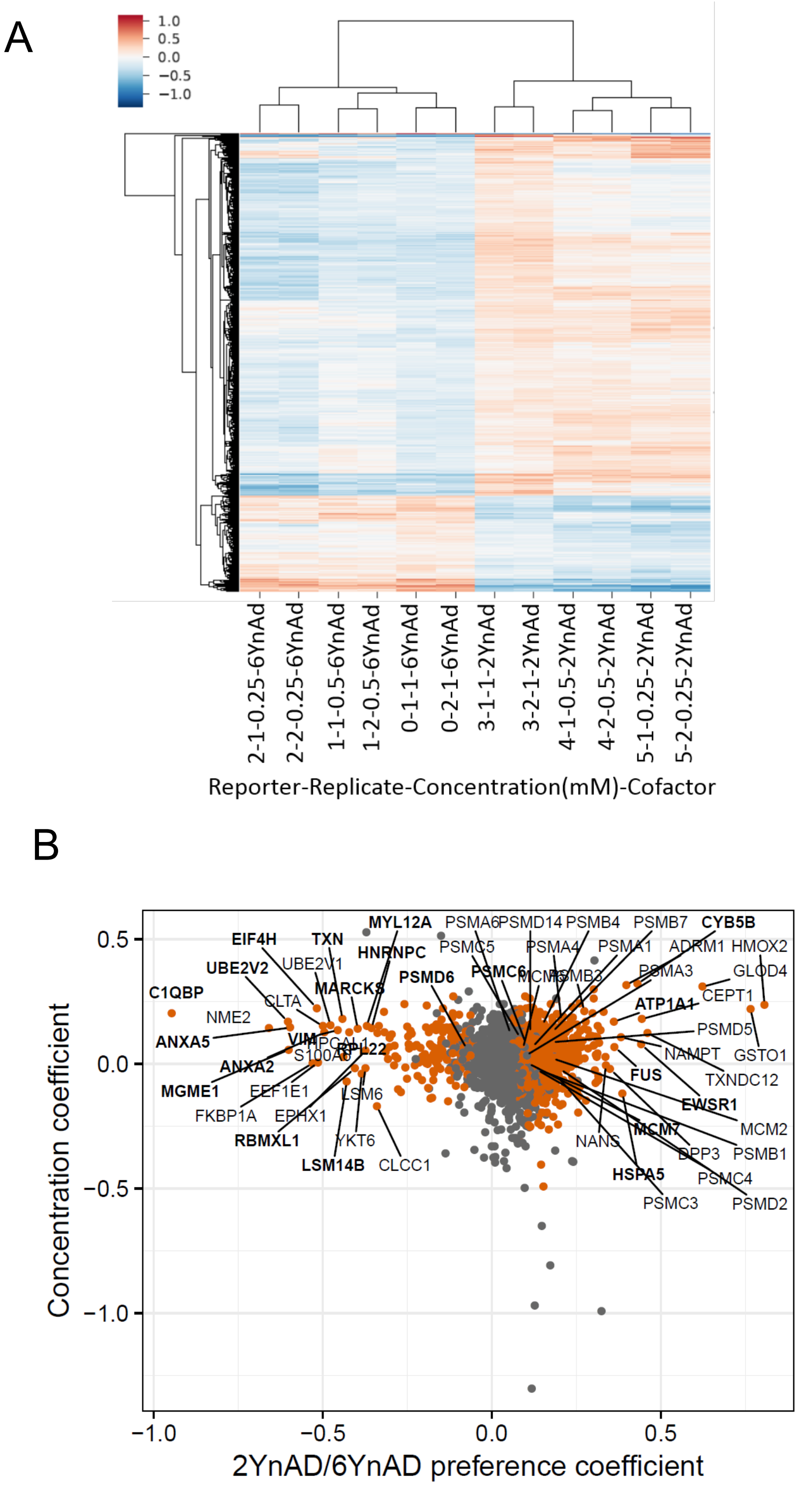

Suppl. Fig. 2. (A) Heatmap indicating mass spectrometry intensities across all three concentrations after subtracting bias terms (e.g. replicate bias and intercept). Red colour indicates higher intensity compared to mean across row. (B) Scatter plot of the value relationship with concentration effect coefficient of the TMTsixplex labelling experiment following LIMMA statistical analysis. Entries in the updated (unpublished) version of the ADPriboDB are highlighted in bold.

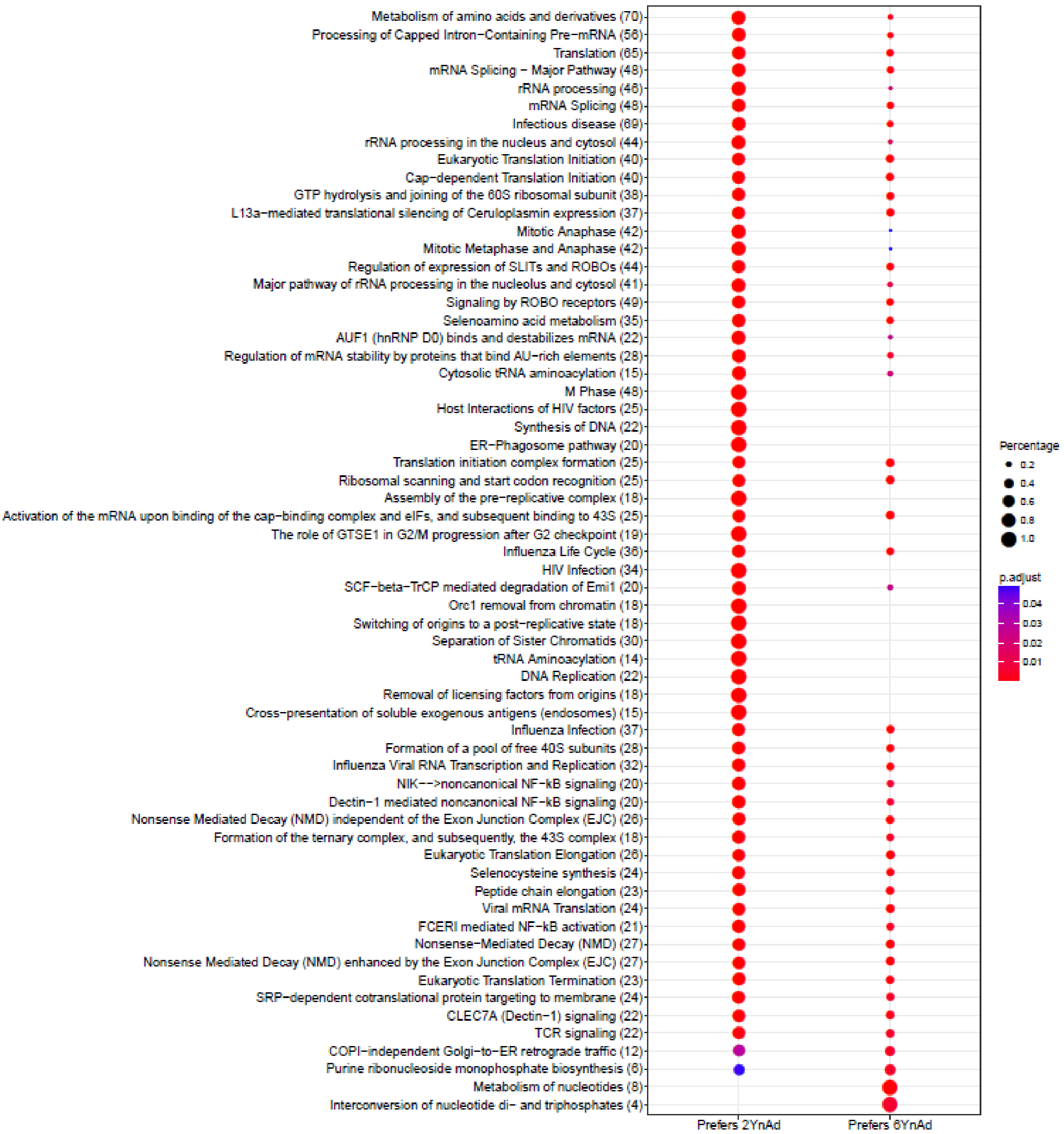

Suppl. Fig. 3. Biological pathway enrichment analysis dot-plots of the preferred targets of 2YnAd and 6YnAd.

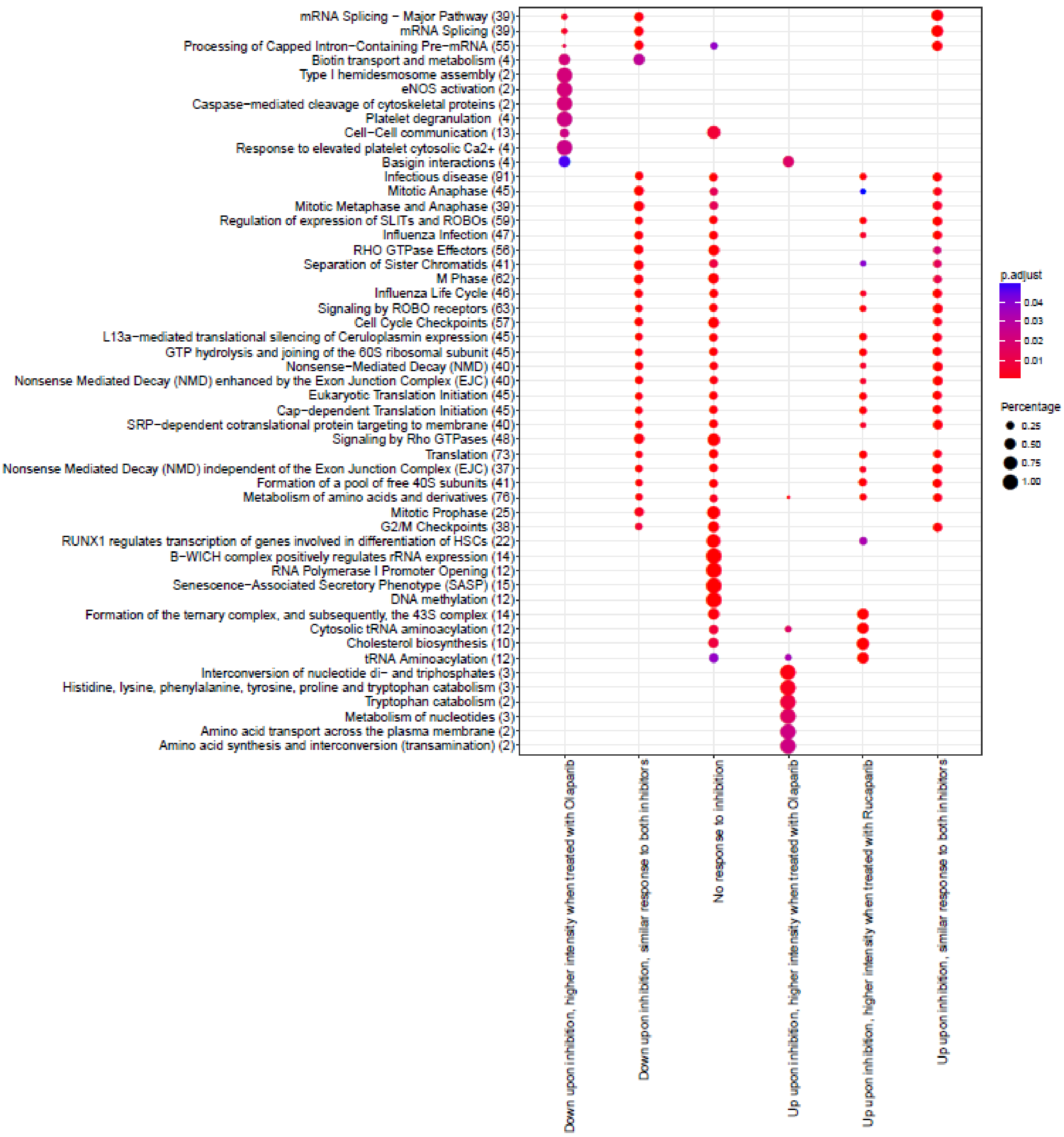

Suppl. Fig. 4. Biological pathway enrichment analysis dot plots of the TMT10plex labelling experiment.

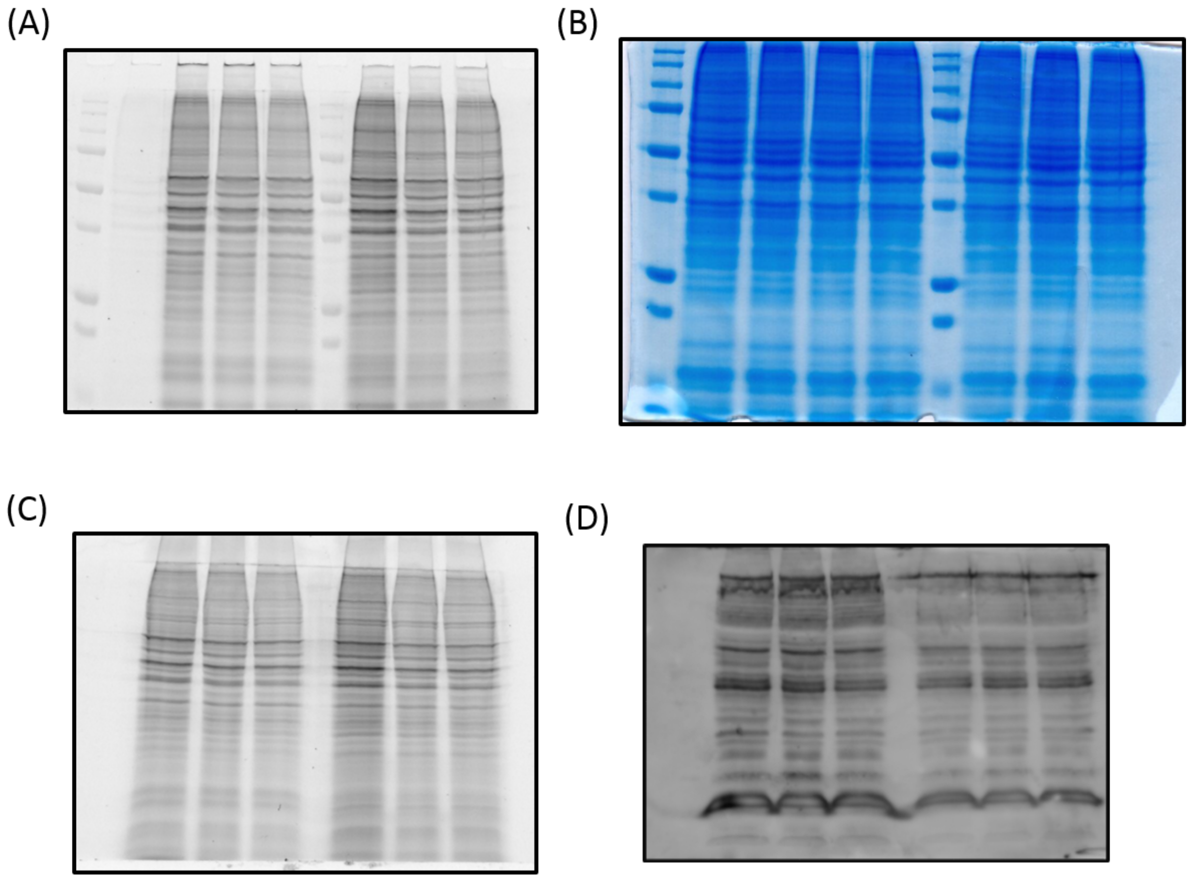

Suppl. Fig. 5. Full-length gels and blot used in Figure 1.

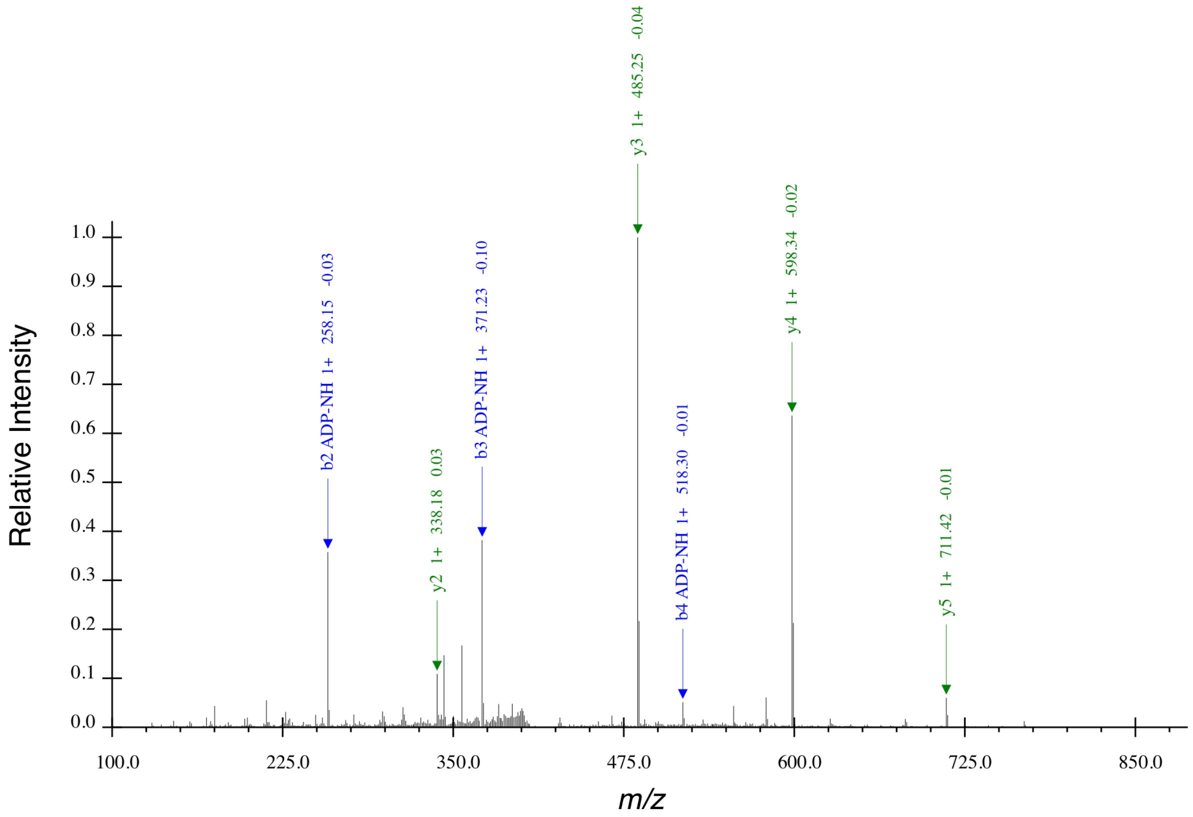

Annotated CID tandem mass spectrum for **E**(NH)ILFYR, z=2, Mp/z^{exact} = 428.2404, Mp/z^{obs} = 428.244, deltaMZ = 0.0037

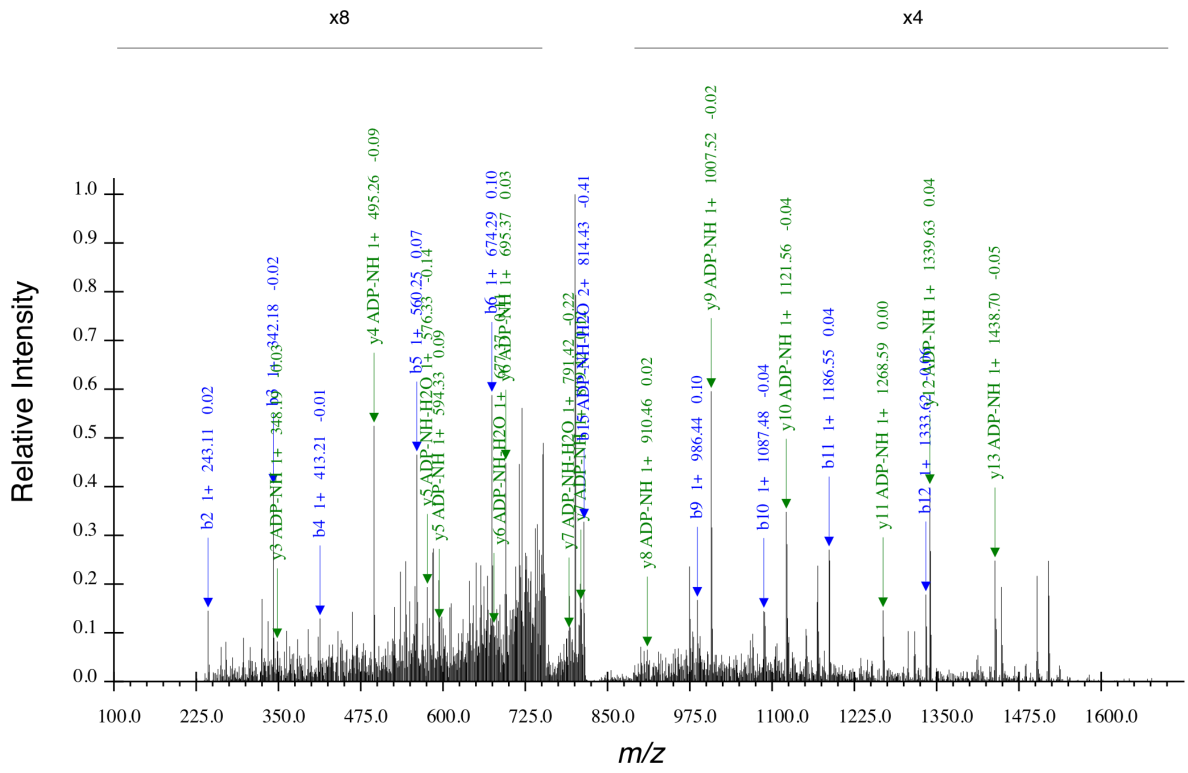

Annotated CID tandem mass spectrum for NQVA**M**(ox)NPTNTVF**D**(NH)AK, z=2, Mp/z^{exact} = 840.9047, Mp/z^{obs} = 840.9014, deltaMZ = -0.0032

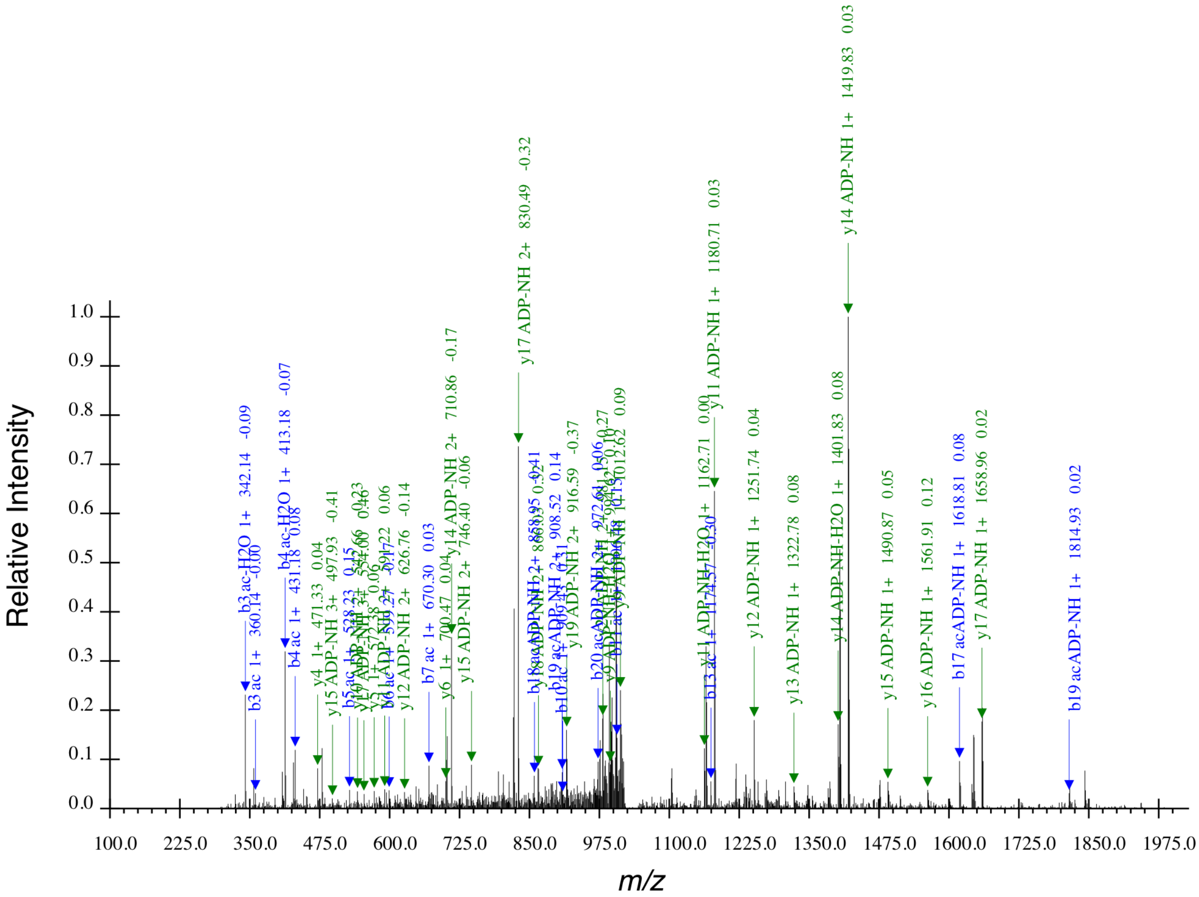

Annotated CID tandem mass spectrum for **ac**-SETAPAAPAAPAPA**E**(NH)KTPVKK,
z=2, Mp/z^{exact} = 1045.0686, Mp/z^{obs} =1045.5692, deltaMZ = 0.5007

Suppl. Fig. 6. Representative MS/MS spectra of modified peptides. Affinity enriched ADP-ribosylated peptides were treated with hydroxylamine to hydrolyse the PARylation to size-reduced, MS-compatible adducts at the modification sites with a characteristic mass signature of +15.0109 Da (the mass of NH). The ADP-ribosylated sites in the tryptic peptide sequence (below each spectrum) are indicated using “NH”.

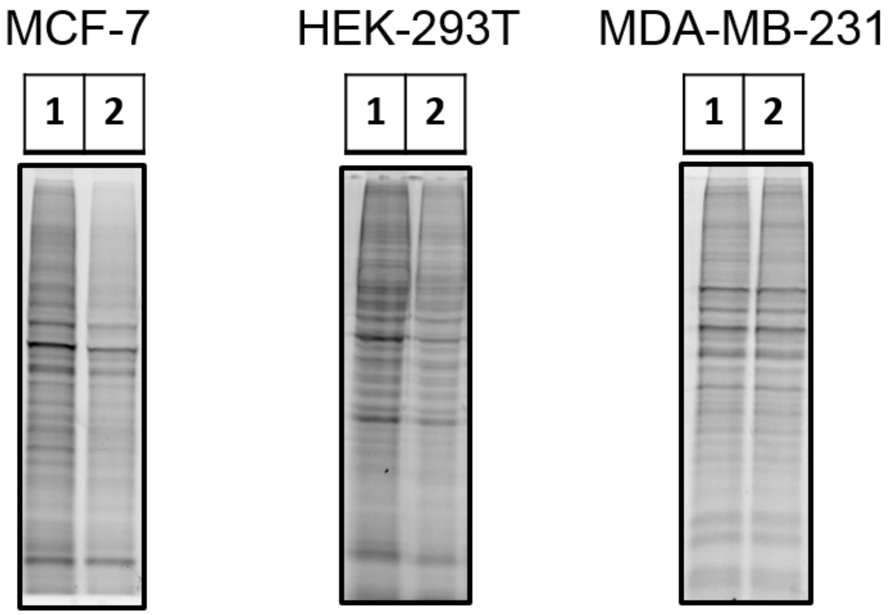

Suppl. Fig. 7. 2YnAd labelling in MCF-7, HEK-293T and MDA-MB-231 cell lines. Lane 1 (0.5 mM) and lane 2 (0.25 mM) probe.

Supplementary Table 1

Design matrix for 2YnAD/6YnAD dataset

Headers				Design matrix			
**Reporter**	**Replicate**	**Concentration_mM**	**Cofactor**	**Intercept**	**C(Cofactor, Sum)[S.2YnAd]**	**C(Replicate)[T.2]**	**np.log(Concentration_mM)**
**0**	**1**	**1**	**6YnAd**	1	-1	0	0
	**2**	**1**	**6YnAd**	1	-1	1	0
**1**	**1**	**0.5**	**6YnAd**	1	-1	0	-0.693147
	**2**	**0.5**	**6YnAd**	1	-1	1	-0.693147
**2**	**1**	**0.25**	**6YnAd**	1	-1	0	-1.386294
	**2**	**0.25**	**6YnAd**	1	-1	1	-1.386294
**3**	**1**	**1**	**2YnAd**	1	1	0	0
	**2**	**1**	**2YnAd**	1	1	1	0
**4**	**1**	**0.5**	**2YnAd**	1	1	0	-0.693147
	**2**	**0.5**	**2YnAd**	1	1	1	-0.693147
**5**	**1**	**0.25**	**2YnAd**	1	1	0	-1.386294
	**2**	**0.25**	**2YnAd**	1	1	1	-1.386294

Supplementary Table 2

Design matrix for PARP inhibitor dataset.

Headers						Design matrix				
**Reporter**	**Replicate**	**Concentration_uM**	**Inhibitor**	**Inhibitor_binary**	**Inhibitor_added**	**C(Inhibitor_binary, contrast)[custom0]**	**C(Inhibitor_added)[T.True]**	**C(Replicate)[T.2]**	**C(Replicate)[T.3]**	**Intercept**
**0**	**1**	25	Olaparib	Olaparib	TRUE	-1	1	0	0	1
	**2**	25	Olaparib	Olaparib	TRUE	-1	1	1	0	1
	**3**	25	Olaparib	Olaparib	TRUE	-1	1	0	1	1
**4**	**1**	0	Olaparib	NA	FALSE	0	0	0	0	1
	**2**	0	Olaparib	NA	FALSE	0	0	1	0	1
	**3**	0	Olaparib	NA	FALSE	0	0	0	1	1
**5**	**1**	25	Rucaparib	Rucaparib	TRUE	1	1	0	0	1
	**2**	25	Rucaparib	Rucaparib	TRUE	1	1	1	0	1
	**3**	25	Rucaparib	Rucaparib	TRUE	1	1	0	1	1
**9**	**1**	0	Rucaparib	NA	FALSE	0	0	0	0	1
	**2**	0	Rucaparib	NA	FALSE	0	0	1	0	1
	**3**	0	Rucaparib	NA	FALSE	0	0	0	1	1
